# Hypermaintenance and hypofunction of aged spermatogonia: insight from age-related increase of Plzf expression

**DOI:** 10.18632/oncotarget.4045

**Published:** 2015-05-08

**Authors:** Ianina C. Ferder, Ning Wang

**Affiliations:** ^1^ Vincent Center for Reproductive Biology, MGH Vincent Department of Obstetrics and Gynecology, Massachusetts General Hospital, Boston, Massachusetts, USA; ^2^ Department of Obstetrics, Gynecology and Reproductive Biology, Harvard Medical School, Boston, Massachusetts, USA

**Keywords:** spermatogonial stem cell, aging, Plzf, mTORC1, differentiation

## Abstract

Like stem cells in other tissues, spermatogonia, including spermatogonial stem cells (SSCs) at the foundation of differentiation hierarchy, undergo age-related decline in function. The promyelocytic leukemia zinc finger (Plzf) protein plays an essential role in spermatogonia maintenance by preventing their differentiation. To evaluate whether there is an age-related change in Plzf expression, we found that aged mouse testes exhibited a robust “Plzf overexpression” phenotype, in that they showed not only a higher frequency of Plzf-expressing cells but also an increased level of Plzf expression in these cells. Moreover, some Plzf-expressing cells in aged testes even aberrantly appeared in the differentiating spermatogonia compartment, which is usually low or negative for Plzf expression. Importantly, ectopic Plzf expression in F9 cells suppressed retinoic acid (RA)-induced Stra8 activation, a gene required for meiosis initiation. These data, together with our observation of a lack of meiosis-initiating spermatocytes associated with high Plzf-expressing spermatogonia in the aged testes, particularly in the degenerative seminiferous tubules, suggest that age-related increase in Plzf expression represents a novel molecular signature of spermatogonia aging by functionally arresting their differentiation.

## INTRODUCTION

Spermatogenesis is an intricate and coordinated process that occurs inside the seminiferous tubules of the testis by which thousands of spermatozoa (sperm) are produced daily from spermatogonia with spermatogonial stem cells (SSCs) at the foundation of the differentiation hierarchy [1, 2]. In mouse, the spermatogonia reside within the basal compartment of the tubule and are classified by morphology into type A_single_, A_paired_, A_aligned_, A_1_, intermediates, and type B [3]. A_single_, A_paired_, and A_aligned_ are considered undifferentiated spermatogonia. Differentiation starts from A_1_ spermatogonia, which undergo five rounds of mitotic division (intermediates) to form B spermatogonia. Spermatogenesis begins shortly after birth. After puberty, spermatogonia undergo both self-renewal and differentiation to maintain the production of sperm, the vehicle by which male genetic information is transmitted to successive generations. The entire developmental process from spermatogonia to spermatozoa takes about 35 days in mice and 64 days in humans and closely interacts with the Sertoli cells, the only somatic cells in the tubules [3]. Outside the tubules, the Leydig cells, nerves, and blood vessels also bring systemic signals that modulate spermatogenesis [1]. Therefore, the balance between these processes is regulated by both specific intrinsic gene expression and extrinsic environmental stimuli. In addition to being the site of spermatozoa production, the testis also serves as an endocrine organ because of the interstitial Leydig cells, which produce testosterone needed for normal spermatogenesis and male phenotypic characteristics [1, 4]. Reciprocally, disruption in spermatogonial activity can also cause abnormal testosterone secretion from the Leydig cells [5], indicating that normal spermatogenesis is important to the entire reproductive axis in males.

Like somatic stem cells, germline stem cells (GSC), such as spermatogonia, undergo age-associated decline in function [6]. For example, there is a dramatic and significant decrease in the numbers of spermatogonia with “stemness” in aged mouse testes [7, 8]. Stemness is defined by the ability to repopulate and form colonies of spermatogenesis upon transplant into young recipient host. These data indicate a decline in the repopulating activity of aged spermatogonia. In addition, altered gene expression is found in the aged spermatogonia, and many of the transcripts are involved in mitosis, DNA damage responses, and oxidative stress, suggesting diminished potential for spermatogonia to alleviate genotoxic stress [9]. However, the molecular mechanism underlying spermatogonia aging is not known. Moreover, since spermatogenesis is a classic stem cell-dependent tissue regeneration process, lessons learned in spermatogonia aging could be valuable in understanding stem cell aging in other tissue-specific systems.

Promyelocytic leukemia zinc finger (Plzf), encoded by *Zbtb16*, is specifically expressed at the spermatogonia stage during spermatogenesis in mouse and human [5, 10, 11]. To date, Plzf has been shown to prevent spermatogonia differentiation by repressing c-Kit expression [12] or by suppressing mTORC1 activity, the master regulator of cell growth [13]. Plzf is indispensible for spermatogonia maintenance in that Plzf deletion leads to spermatogonia depletion and results in a “testicular aging”-like phenotype with increased numbers of degenerative tubules devoid of germ cells in young adult males [5, 10]. Hence, in this study, we examined whether there is an age-associated loss of Plzf expression in mouse testes. Interestingly, we found that aged testes exhibited a robust “Plzf overexpression” phenotype, which probably causes hypermaintenance of spermatogonia that contributes to their decline in function.

## RESULTS

### Plzf expression is significantly elevated in aged testes

**Figure 1:**
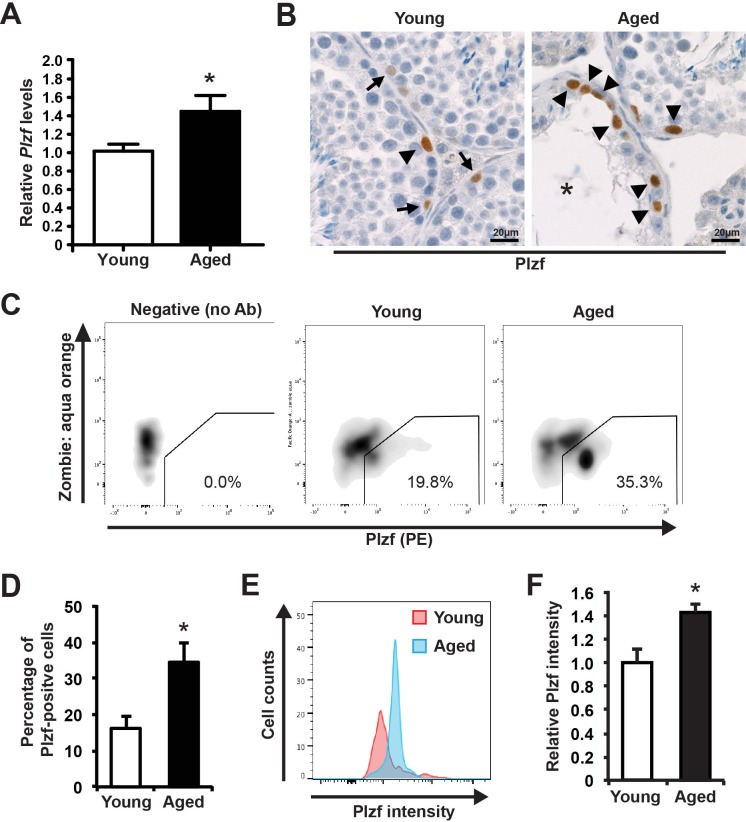
Plzf expression is elevated in aged mouse testes **A**, qPCR analysis of *Plzf* mRNA in young and aged testes normalized to *Ddx4*. Graphs represent mean value ± s.e.m. n = 9 mice per group. *P < 0.05. **B**, representative images of IHC for Plzf in young and aged testes. Images are representative of testes from 3 mice examined. Arrow, low Plzf. Arrowhead, high Plzf. *, degenerative seminiferous tubule. **C**, representative flow profile of detecting Plzf-expressing cells by intracellular staining and FACS by using anti-Plzf antibody (PE-conjugated). **D**, quantification of the percentage of Plzf-positive cells analyzed by FACS in panel C. Graphs represent mean value ± s.e.m. n = 6 mice per group. *P < 0.05. **E**, Plzf intensity in Plzf-expressing cells from young versus aged mice. **F**, Quantification of Plzf expression levels in young and aged testes. Graphs represent mean value ± s.e.m. n = 4 mice per group. *P < 0.05.

We collected seminiferous tubules from young (3 month old) and aged (24 month old) mice and conducted qRT-PCR analysis for *Plzf*. To our surprise, we found that *Plzf* mRNA was significantly elevated in aged testes when compared with that of young testes (Figure 1A and Supplementary Figure 1). Further immunohistochemistry (IHC) staining for Plzf in testicular cross sections revealed that, while in young testes there were differential levels of Plzf expression with Plzf-high (arrowhead in Figure 1B) and Plzf-low cells (arrows in Figure 1B), there were more cells showing high levels of Plzf expression in aged testes and many of these Plzf-high cells accumulated in the degenerated tubules where spermatogenesis was already ceased (Figure 1B). In fact, 83.9% of degenerated tubules (26 out of 31 examined from 4 aged testes) showed Plzf-expressing cells. To provide a quantitative analysis of Plzf expression, we used flow cytometry (FACS)-based intracellular staining to quantify the frequencies of Plzf-expressing cells in dissociated testes (Figure 1C). We found that aged testes showed a 2.1-fold increase in the frequency of Plzf-expressing cells when compared with that of young testes (Figure 1D). Additionally, consistent with our histological analysis (Figure 1B), Plzf-expressing cells in aged testes exhibited a higher intensity of Plzf expression than those in young testes (Figure 1E and 1F).

### Spermatogonia in aged testes show decline in their differentiation activity

**Figure 2:**
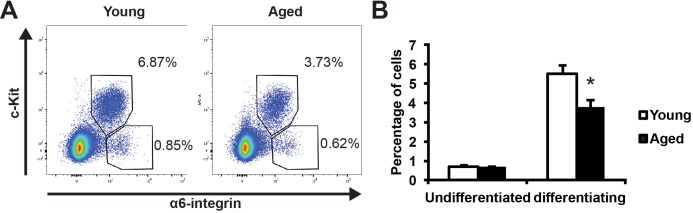
Comparison of spermatogonial differentiation in young and aged mouse testes **A**, representative profiles of undifferentiated (c-Kit-negative α6-integrin-high) and differentiating (c-Kit-positive α6-integrin-low) spermatogonia from young (left) and aged (right) testes. **B**, quantification of the percentages of the undifferentiated and differentiating spermatogonia in young versus aged testes analyzed by FACS in panel A. Graphs represent mean value ± s.e.m. n = 5 samples per group. *P < 0.05.

We used an established strategy to quantitatively analyze spermatogonial development by using two cell surface markers: α6-integrin and c-Kit [14, 15]. α6-integrin marks undifferentiated spermatogonia with stem cell capacity, and c-Kit marks differentiating spermatogonia [16, 17]. This allowed us to separate two distinct populations by FACS as carefully characterized by previous study [14]: the c-Kit-negative α6-integrin-high population that is enriched for undifferentiated spermatogonia and the c-Kit-positive α6-integrin-low population that is enriched for differentiating spermatogonia (Figure 2A). By comparing the profiles of these two spermatogonia populations, we found that the aged testes showed a comparable frequency (0.652% vs. 0.716%, *P* = 0.3) of undifferentiated spermatogonia but significantly lowered percentage of differentiating spermatogonia (3.80% vs. 5.46%, *P* < 0.05) when compared with young testes (Figure 2B). These data suggest that there is a decline of spermatogonial differentiation activity in aged testes.

### Plzf is aberrantly expressed in the differentiating (c-Kit-positive) spermatogonia in aged testes

**Figure 3:**
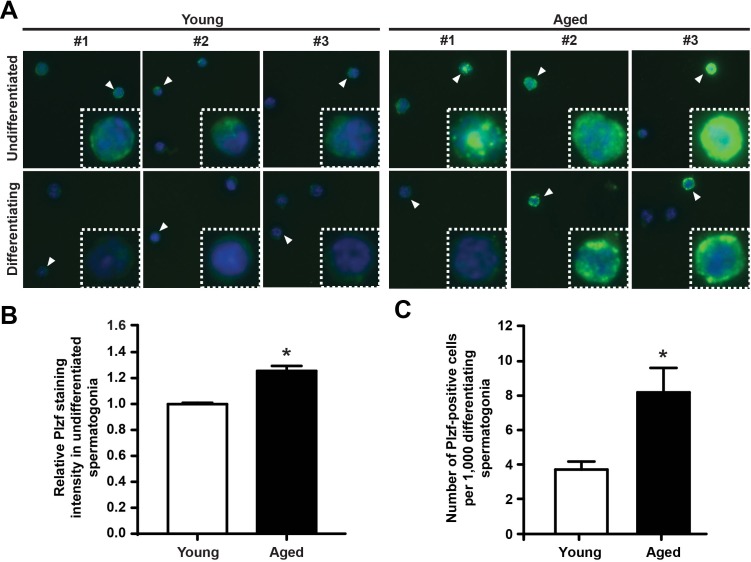
Plzf is expressed in differentiating spermatogonia from aged mouse testes **A**, IF staining for Plzf in isolated undifferentiated and differentiating spermatogonia from young and aged mice. Data shown are representative images from 3 mice for each group and 200 cells from each mouse. **B**, quantitative analysis of Plzf immunofluorescence staining intensity of the c-Kit-negative α6-integrin-high undifferentiated spermatogonia from young and aged testes. *P < 0.01, n = 93 – 98 cells from 3 – 4 mice per group. Plzf staining intensity in all cells from 3 or 4 different areas per each mouse were measured. **C**, numbers of Plzf-expressing cells within the c-Kit-positive α6-integrin-low differentiating spermatogonia compartment in young and aged testes. Graphs represent mean value ± s.e.m. * *P* < 0.05, n = 4 mice per group.

To compare the cellular expression pattern of Plzf between young and aged testes, we next isolated the undifferentiated and differentiating spermatogonia from testes of young and aged mice by FACS (as shown in Figure 2A) and sorted these cells directly onto poly-D-lysine-coated slides. To ensure comparable results, cells from each group were spotted at different spots on the same slide. We next conducted immunofluorescence staining for Plzf on these samples. Consistent with our previous observation by FACS or by detecting Plzf *in situ* using IHC (Figure 1), we found a markedly elevated level of Plzf expression in the undifferentiated spermatogonia isolated from aged mice compared with those from young mice (Figure 3A, upper panel, and 3B). Additionally, in contrast to the differentiating spermatogonia from young mice that only showed weak or no Plzf staining, some differentiating spermatogonia from aged testes were clearly positive for Plzf (Figure 3A, lower panel). Quantitative analysis further confirmed a significant increase in the number of Plzf-expressing differentiating spermatogonia in aged testes compared with that in young testes (Figure 3C).

### Plzf upregulation correlates with low mTORC1 activity in aged testes

**Figure 4:**
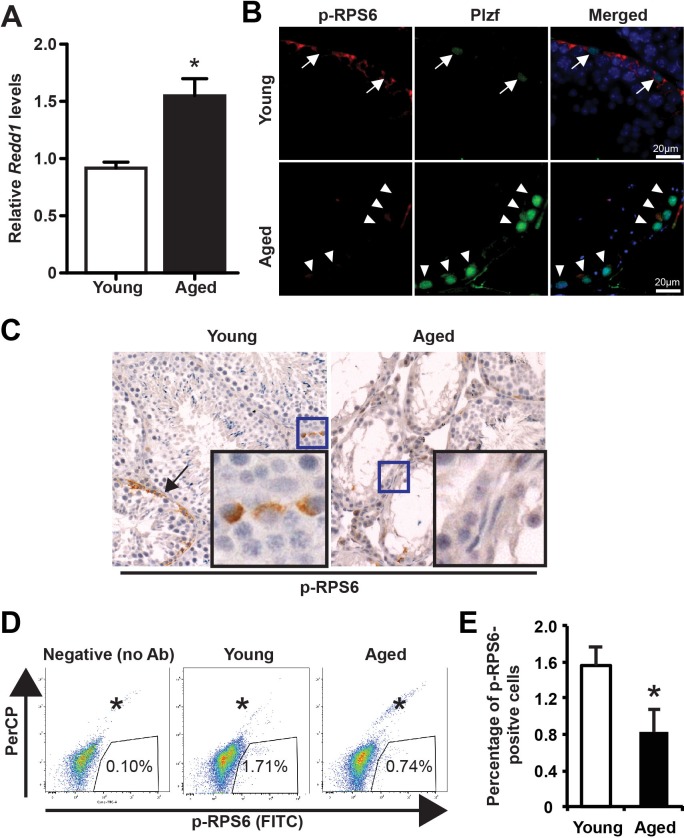
Low mTORC1 activity in aged testes **A**, qPCR for *Redd1* mRNA in young versus aged testes normalized to *β-actin*. Graphs represent mean value ± s.e.m. n = 6 mice per group. *P < 0.05. **B**, dual-immunofluorescence staining for Plzf and p-RPS6 in young (upper) and aged (lower) testes. Arrow, low Plzf. Arrowhead, high Plzf. **C**, IHC for p-RPS6. Data shown are testes from 3 separate mice each group. **D**, representative flow profile of detecting p-RPS6 by using rabbit anti-p-RPS6 antibody (Alexa fluor 488-conjugated). **E**, quantification of the percentage of p-RPS6-positive cells analyzed by FACS in panel D. Graphs represent mean value ± s.e.m. n = 4 samples per group. 2 mice per group *P < 0.05.

It has been reported that Plzf suppresses mTORC1 activity by transcriptionally activating *Redd1* to maintain spermatogonia self-renewal [13]. To determine whether there is an age-associated change in this pathway, we found that *Redd1* mRNA was significantly elevated in aged testes (Figure 4A). Next, to determine whether mTORC1 activity is decreased in association with Plzf overexpression in aged spermatogonia, we measured the phosphorylation of ribosomal protein S6 (p-RPS6), which is a downstream target of mTORC1 through ribosomal protein S6 Kinase 1 [18, 19]. In dual-immunofluorescence staining for p-RPS6 (Ser235/236) and Plzf, we found that in young testes Plzf-expressing cells were surrounded by p-RPS6 cells, which probably are their differentiating progeny (Figure 4B). However, there was a lack of p-RPS6 expression associated with strong Plzf expression in aged testes, particularly in the degenerative seminiferous tubules (Figure 4B). Histological analysis of IHC staining for p-RPS6 confirmed an overall lack of p-RPS6 expression in aged testes (Figure 4C). Furthermore, we used FACS-based intracellular staining to measure p-RPS6 expression (Figure 4D). Aged testes showed a 53.0% reduction in the percentage of p-RPS6-positive cells when compared with young testes (34.5% vs. 16.2%, *P* < 0.5) (Figure 4E). Additionally, aged testes showed enhanced GDNF receptor expression (Supplementary Figure 2), similar to prolonged pharmacological inhibition of mTORC1 activity by rapamycin treatment [13].

### Ectopic Plzf expression in F9 cells prevents retinoic acid (RA)-induced Stra8 activation

**Figure 5:**
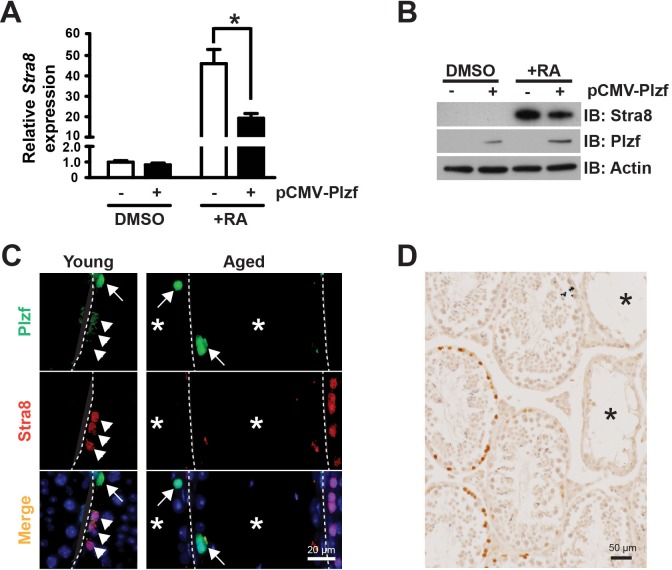
Plzf expression inhibits RA-induced Stra8 transcription in F9 cells and correlates with a lack of Stra8-expressing cells in aged testes **A**, qPCR analysis for *Stra8* mRNA in F9 cells normalized to *β-actin*. Graphs represent mean value ± s.e.m. n = 3 independent cultures per group. *P < 0.05. **B**, western blotting analysis of Stra8 in F9 cells. Data are representative of 2 independent sets of experiments. **C**, dual-IF staining for Plzf and Stra8 in young (left) and aged (right) testes. Arrow, Plzf-expressing cells. Arrowhead, Stra8-expressing cells. **D**, IHC for Stra8 in aged testes. *, degenerative seminiferous tubule.

To provide function insight into the consequence of Plzf overexpression observed in aged testes, we examined the impact of ectopic Plzf expression on *stimulated by retinoic acid gene 8* (*Stra8*) transcription in F9 cells. by transient transfection *Stra8* activation is among the earliest events known to date during meiosis initiation [20, 21]. F9 cells are embryonic carcinoma cells that activate *stimulated by retinoic acid gene 8* (*Stra8*) transcription in response to retinoic acid (RA) stimulation [22-24]. Therefore, F9 cells have been widely used to study *Stra8* transcription [25, 26]. Ectopic Plzf expression significantly inhibited RA-induced *Stra8* activation at mRNA level (Figure 5A) as well as reduced Stra8 expression at protein level (Figure 5B). To confirm such findings, we observed that, unlike young testes, there was a lack of Stra8-expressing, meiosis-initiating spermatocytes associated with Plzf-expressing spermatogonia in the aged testes, particularly in the degenerative seminiferous tubules (Figure 5C). In fact, despite that 83.9% of degenerative tubules in aged testes contained Plzf-expressing spermatogonia, 91.4% of them (32 out of 35 examined in 4 testes) did not show Stra8-expressing preleptotene spermatocytes entering meiosis (Figure 5D). These data suggest that age-related increase in Plzf expression may impede spermatogonia differentiation and thus prevent meiosis initiationi a condition that occurs in aged testes as reflected by a decreased percentage of α6-integrin-low c-Kit-positive differentiating spermatogonia (Figure 2).

## DISCUSSION

**Figure 6:**
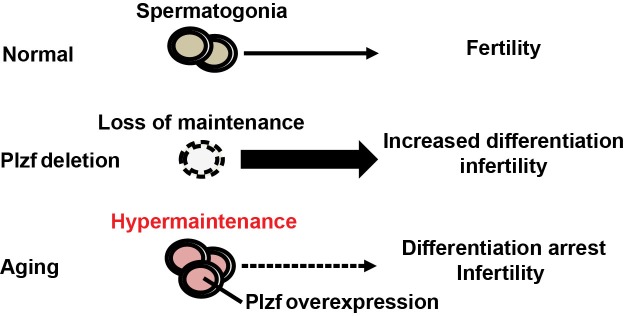
Schematics of Plzf overexpression and spermatogonia aging Loss of Plzf exhausts spermatogonia pool by promoting their differentiation at the expense of self-renewal. On the contrary, age-related increase in Plzf expression causes spermatogonia hypermaintenance and aging by preventing their differentiation.

The study characterized Plzf overexpression as a novel molecular signature of spermatogonia aging using mouse testis as a model. This is consistent with the previous report that *Plzf* is among the genes enriched in aged rat spermatogonia by gene array analysis [9]. Robust “Plzf overexpression” phenotype observed in aged mouse testes, which showed not only a higher frequency of Plzf-expressing cells but also an increased level of Plzf expression in these cells (Figure 1). Moreover, some Plzf-expressing cells in aged testes even aberrantly appeared in the differentiating spermatogonia compartment, which is usually low or negative for Plzf expression (Figure 3). To examine the functional consequence of Plzf overexpression, we found that aged testes showed a decreased level of mTORC1 activity compared to that in young testes (Figure 4). Importantly, ectopic Plzf expression in cultured F9 cells was sufficient to prevent RA-induced *Stra8* activation, a gene required for meiosis initiation (Figure 4). This is consistent with our observation of a lack of meiosis-initiating spermatocytes associated with Plzf-expressing spermatogonia in the aged testes, particularly in the degenerative seminiferous tubules (Figure 5). Hence, contrary to Plzf deletion, which results in the lack of spermatogonia maintenance and thereby promotes their differentiation, age-related increase in Plzf expression may actually result in the hypermaintenance of spermatogonia by preventing their normal differentiation and cause a decline in regenerative function (Figure 6).

### Plzf overexpression-mediated inhibition of spermatogonial function

Given that Plzf deletion results in spermatogonia depletion due to differentiation at the cost of maintenance [5, 10], it is probably not surprising to see that age-related increase in Plzf expression in spermatogonia, on the contrary, suppresses their differentiation. In addition, Plzf has been reported to function as an inhibitor of differentiation in neural tissues [27]. Mechanistically, RA-induced transcriptional activation of *Stra8* through RA receptors, the RARs, is probably an early event known to date during spermatogonial differentiation [21]. Plzf has been reported to suppress the transcription activity of RARs [28]. Therefore, in addition to suppress mTORC1 activity via Redd1 [13], age-related increase in Plzf expression may also interfere with the ability of RARs to activate *Stra8*, and thereby prevent spermatogonia differentiation at the stage of meiotic initiation.

In addition, Ryu *et al* described that there was a dramatic and significant age-associated decline in the number of spermatogonia with stemness per testis [7]. Stemness is reflected by the capacity of spermatogonia to repopulate chemotherapy-depleted testes. In our current study, we find that Plzf-expressing spermatogonia exist in aged testes at an even higher frequency when compared to that in young testes. Therefore, the numbers of spermatogonia do not decrease in aged testes, but, instead, it seems that some of these spermatogonia might lose their stemness with advancing age. Hence, it would be interesting to determine whether Plzf overexpression has a negative impact on the stemness of spermatogonia.

### mTORC1 – longevity and reproduction

Pharmacological mTOR inhibition extends lifespan [29-33]. However, here we show that age-related increase in Plzf expression may function as a natural inhibitor of mTORC1 activity during aging (Figure 4). Hence, activation of mTOR signaling is required for gametogenesis and reproduction. These data recall the prevailing theory that reproduction is at the cost of lifespan. Thus, further understanding of spermatogonia aging is needed to devise targeted therapy to effectively delay reproductive aging without compromising pathways involved in controlling lifespan.

### Extrinsic regulators of Plzf

Spermatogonia undergo age-related decline in function like somatic stem cells. However, unlike somatic stem cells, spermatogonia as GSCs may stay “forever young” intrinsically. For example, telomerase activity in aged spermatogonia remains high despite their attenuated function in aged testes [22]. More strikingly, although aged spermatogonia show decreased colony-forming activity at the initial transplant into young recipient testes, their activity appears to be rejuvenated upon engrafting into the young recipient microenvironment and can be maintained after being serially transplanted into young recipient hosts for over 3 years without any decline in function [7]. Thus, it is the deterioration of the microenvironment in which spermatogonia reside that triggers their declining activity with age through intrinsic alterations [7, 9], e.g, regulation of Plzf expression (Supplementary Figure 3). This notion is further supported by studies of GSCs in *Drosophila*. For example, a decline in paracrine factors that stimulate GSC self-renewal [34] or a loss of anchorage proteins that bridge GSCs with niche cells occurs in gonads of aged but not young flies [35] – both have been attributed to decreased GSC activity with age.

Taken together, given the critical roles of Plzf in normal spermatogonia development and its overexpression phenotype in aged testes, future studies on characterizing the age-related alterations of extrinsic cues emanated from the microenvironment that causes Plzf upregulation will be critical to understand spermatogonia aging.

## MATERIALS AND METHODS

### Animals

Male 3 month old (young) and 24 month old (aged) C57Bl/6 mice were ordered from NIA. Mice were euthanized and their testes were isolated for experiments. All procedures and care of animals were carried out according to the Massachusetts General Hospital Institutional Animal Care and Use Committee (IACUC).

### Isolation of mouse testicular germ cells and FACS analysis

Testes were collected and decapsulated. To minimize the contamination of interstitial tissues, e.g., Leydig cells, seminiferous tubules were washed three times in Hank's Balanced Salt Solution (HBSS) and allowed to sediment naturally. Testis cell suspensions were then generated by sequential digest of dissected and minced seminiferous tubules with 1 mg/ml Type IV collagenase (Worthington) in the presence of 10 μg/ml DNAse I (Sigma) and 0.05% Trypsin (Gibco). Trypsin was neutralized by 10% fetal bovine serum (FBS). Cells were passed through a 70 μm strainer to remove clumps. Spermatogonia were resuspended in HBSS containing 0.2% FBS for subsequent staining. The following antibodies to cell surface markers were used: APC-conjugated c-Kit antibody (CD117, Bioscience Catalog #553356) and PE-conjugated α6-integrin (CD49f, Bioscience Catalog #555736). For FACS analysis of Plzf staining, FoxP3 staining kits (eBioscience) were used according to manufacturer's instructions to permeabilize the cells. All samples were diluted at the same cell density and then stained at the same antibody dilution by PE anti-mouse Plzf Antibody (clone 9E12, Biolegend Catalog #145804). For FACS analysis of p-RPS6 staining, we fixed equal numbers of testicular cells from young and aged mice in 2% formaldehyde at 37ÐC for 20 min and then permeabilize these cells with 90% methanol. After washing the cells with Phosphate-buffered saline (PBS), we blocked the cells with PBS containing 1% BSA for 10 min and then incubate the cells with Alexa fluor 488-conjugated p-RPS6 (Ser235/236, clone 2F9, Cell Signaling Technology Catalog #4854) for 30 min. Cells were washed with PBS 3 times again and subject to FACS analysis.

### Immunohistochemistry and immunofluorescence

Testes were fixed in 4% paraformaldehyde, embedded in paraffin and sectioned for analysis. Antibodies used include: Plzf (clone D-9, Santa Cruz Biotechnology Catalog# 28319), Stra8 (ab49602, abcam), and p-RPS6 (Ser235/236). For immunofluorescence, detection was performed using Alexa fluor 488-conjugated donkey anti-mouse and Alexa fluor 546-conjugated donkey anti-rabbit secondary antibodies. For immunohistochemistry, detection was performed using goat anti-mouse or goat anti-rabbit as secondary antibody for horseradish peroxidase-based DAB detection (DAKO). For immunocytochemistry, cells were sorted directly onto poly-Lysine-D-coated slides. Cells were then fixed in 4% PFA for 15 minutes before staining. Images were captured using a Nikon ECLIPSE TE2000-S microscope and were analyzed by Image J software (National Institutes of Health).

### Cell culture and plasmid

F9 germline cells (ATCC CRL-1720; American Type Culture Collection) were grown at 37 °C in a humidified atmosphere of 5% CO_2_-95% air on 0.1% gelatin-coated tissue culture plates in DMEM containing 4.5 g/l glucose and supplemented with 10% FBS (Invitrogen), 2 mM L-glutamine, 50 μg/ml penicillin and 50 μg/ml streptomycin. Cells were transfected using lipofectamine (Invitrogen) and were starved for 24 hours in 10% charcoal-stripped FBS before being treated with all-trans RA (Sigma) at 1 μM for 24 hours. Plzf expression plasmid was obtained from Origene (RC206745).

### Gene expression analysis

One μg of total RNA from each sample was reverse transcribed by using SuperScript III from Invitrogen. qPCR was conducted by using SsoAdvanced™ Universal SYBR® Green Supermix with the following primers:

*Plzf* forward, 5′-AAACGGTTCCTGGACAGTTTGCGAC-3′
reverse, 5′-CCAGTATGGGTCTGTCTGTGTGTCTCC-3′
*Redd1* forward, 5′-CAAGGCAAGAGCTGCCATAG-3′,
reverse, 5′-CCGGTACTTAGCGTCAGGG-3′;
*Stra8* forward, 5′-CCTGGTAGGGCTCTTCAACA-3′
reverse, 5′-GGCTTTCTTCCTGTTCCTGA-3′
*Gfra1* forward, 5′-CACTCCTGGATTTGCTGATGT-3′
reverse, 5′-AGTGTGCGGTACTTGGTGC-3′
*c-Ret* forward, 5′-GCATGTCAGACCCGAACTGG-3′
reverse, 5′-CGCTGAGGGTGAAACCATCC-3′
*β-actin* forward, 5′-CTGCCGCATCCTCTTCCTC-3′
reverse, 5′-GCCACAGGATTCCATACCCA-3′
*Ddx4* forward, 5′-AACCTACCGTTGGAGCAGATA-3′
reverse, 5′-TTGTGACATTGAGCTTGTCGT-3′
*Dazl* forward, 5′-TCTGCCACAACTTCTGAGGC-3′
reverse, 5′-CCTGATTTCGGTTTCATCCATCC-3′

### Western blotting analysis

Total protein was isolated in RIPA buffer supplemented with 1 mM PMSF (Sigma) and a protease inhibitor cocktail (Sigma P8340). Lysates were cleared by centrifugation at 14,000 × *g* for 10 min at 4 °C, and protein concentrations in supernatants were determined (DC protein assay; BioRad). Equal amount of protein from each sample was mixed with LDS sample buffer (Invitrogen) plus sample reducing agent (Invitrogen), and denatured for 10 min at 70°C. Proteins were resolved in 4−12% Bis-Tris gels (Invitrogen), and transferred to PDVF membranes. Blots were probed with antibodies against Stra8 (Abcam Catalog# ab49602), Plzf, or pan-Actin (NeoMarkers Catalog# MS-1295), washed and reacted with horseradish peroxidase-conjugated goat anti-rabbit or anti-mouse IgG (BioRad). Detection was performed with the Clarity™ ECL Western Blotting Substrate (BioRad).

### Data analysis and presentation

All experiments were independently replicated at least three times, using different mice, tissues or cells for each replicate. Where possible, assignment of mice to experimental groups was made randomly. Quantitative data from replicate experiments were pooled, analyzed by ANOVA, Mann-Whitney test, or Student *t*-test in GraphPad Prism software, and are presented as the means ± s.e.m. Representative images obtained from immunohistochemical and immunofluorescence assays are provided for qualitative assessment.

## SUPPLEMENTARY MATERIALS FIGURES


