# Supplementary material for: Hypermaintenance and hypofunction of aged spermatogonia: insight from age-related increase of Plzf expression
Source: Oncotarget. 2015 May 8;6(18):15891–901. doi: 10.18632/oncotarget.4045 (PMC4599245; doi:10.18632/oncotarget.4045)
Supplement: Supplementary file 1 [file oncotarget-06-15891-s001.pdf]

Hypermaintenance and hypofunction of aged spermatogonia: insight from age-related increase of Plzf expression

Supplementary Material

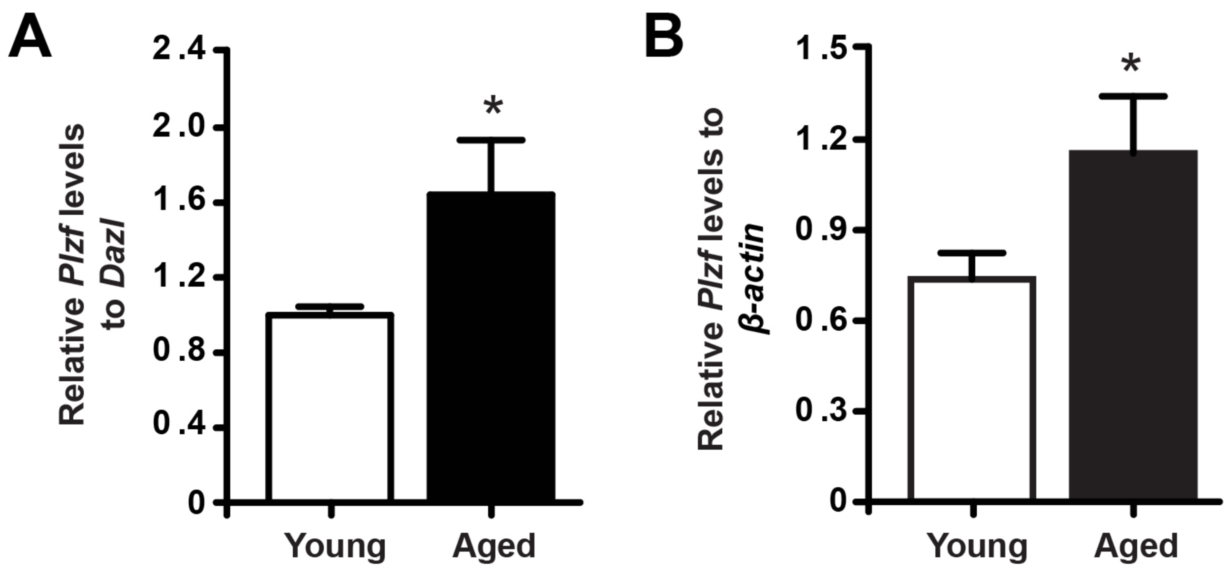

Supplementary Figure 1. *Plzf* expression is elevated in aged mouse testes. qPCR analysis of *Plzf* in young and aged testes normalized to *Dazl* (**A**) or *β-actin* (**B**). Graphs represent mean value ± s.e.m. n = 8 - 12 mice per group. *$P < 0.05$.

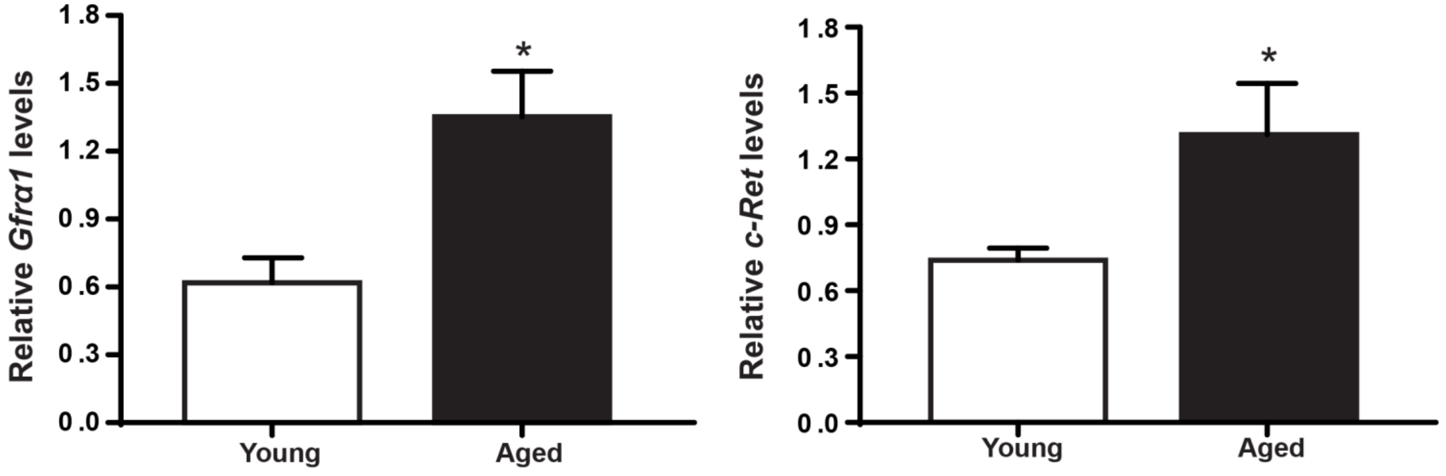

Supplementary Figure 2. GDNF receptor expression is elevated in aged mouse testes. qPCR analysis of *Gfra1* (left) and *c-Ret* (right) in young and aged testes normalized to *β-actin*. Graphs represent mean value ± s.e.m. n = 6 mice per group. *$P < 0.05$.

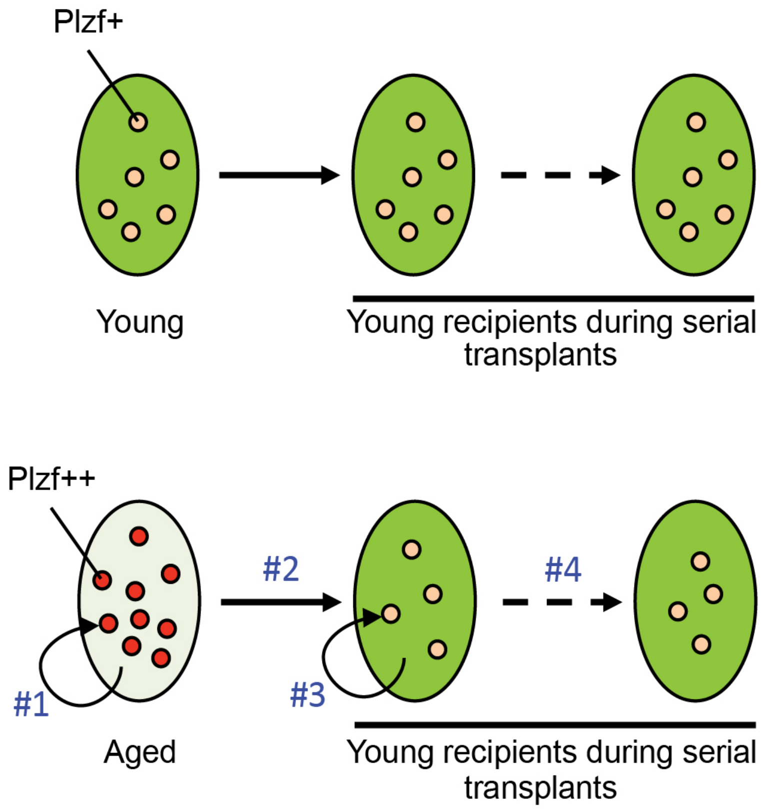

Supplementary Figure 3. Schematics of extrinsic factor-induced intrinsic aging of spermatogonia.

1. Aged microenvironment triggers intrinsic alterations in spermatogonia. In this study, we describe “Plzf overexpression” as a possible molecular signature that negatively affects spermatogonial activity.

2. Spermatogonia freshly isolated from aged testes show decline in their stemness or repopulating potential. Therefore, these aged spermatogonia form fewer number of colonies upon transplant into young recipient testes.

3. Since extrinsic cues cause the intrinsic defects of spermatogonia, engraftment of aged spermatogonia into young microenvironment correct their intrinsic alterations. Aged spermatogonia are rejuvenated!

4. Aged spermatogonia can be serially transplanted into young recipient testes for 3+ years without significant loss of activity, since the young microenvironment keeps them young.
